# Volar locking plate versus external fixation for unstable dorsally displaced distal radius fractures–A 3-year cost-utility analysis

**DOI:** 10.1371/journal.pone.0240377

**Published:** 2020-10-08

**Authors:** Jenny Saving, Emelie Heintz, Hans Pettersson, Anders Enocson, Cecilia Mellstrand Navarro

**Affiliations:** 1 Department of Clinical Science and Education, Södersjukhuset, Karolinska Institutet, Stockholm, Sweden; 2 Capio Artro Clinic, Stockholm, Sweden; 3 Department of Learning, Informatics, Management and Ethics (LIME), Karolinska Institutet, Stockholm, Sweden; 4 Department of Molecular Medicine and Surgery, Karolinska University Hospital, Karolinska Institutet, Stockholm, Sweden; 5 Department of Hand Surgery, Södersjukhuset, Stockholm, Sweden; University of Otago Division of Health Sciences, NEW ZEALAND

## Abstract

**Aim:**

To investigate the cost-effectiveness of Volar Locking Plate (VLP) compared to External Fixation (EF) for unstable dorsally displaced distal radius fractures in a 3-year perspective.

**Methods:**

During 2009–2013, patients aged 50–74 years with an unstable dorsally displaced distal radius fracture were randomised to VLP or EF. Primary outcome was the incremental cost-effectiveness ratio (ICER) for VLP compared with EF. Data regarding health effects (Quality-adjusted life years, QALYs) was prospectively collected during the trial period until 3 years after surgery. Cost data was collected retrospectively for the same time period and included direct and indirect costs (production loss).

**Results:**

One hundred and thirteen patients (VLP n = 58, EF n = 55) had complete data until 3 years and were used in the analysis. At one year, the VLP group had a mean incremental cost of 878 euros and a gain of 0.020 QALYs compared with the EF group, rendering an ICER of 43 900 euros per QALY. At three years, the VLP group had a mean incremental cost of 1 082 euros and a negative incremental effect of -0.005 QALYs compared to the EF group, which means that VLP was dominated by EF. The probability that VLP was cost-effective compared to EF at three years, was lower than 50% independent of the willingness to pay per QALY.

**Conclusion:**

Three years after distal radius fracture surgery, VLP fixation resulted in higher costs and a smaller effect in QALYs compared to EF. Our results indicate that it is uncertain if VLP is a cost-effective treatment of unstable distal radius fractures compared to EF.

## Introduction

The incidence of surgical treatment of distal radius fractures has increased since the introduction of the volar locking plate (VLP) at the turn of the 21st century [1]. VLP has become the most commonly used surgical method, while the use of percutaneous methods, i.e. percutaneous pinning or external fixation (EF), has been reported to decrease [1–3]. There is little evidence to support that any surgical method yields superior clinical outcome as compared to others for treatment of distal radius fractures [4–6]. Other factors than final clinical outcome may therefore be allowed to influence treatment method choices. In a setting with limited health care resources, cost-effectiveness of different methods may be an important aspect to address in the choice of treatment, i.e. if the surgical methods have a reasonable incremental cost in relation to their effects. There is some evidence suggesting that VLP is not a cost-effective surgical technique when compared to percutaneous pinning [7,8]. To the best of our knowledge, health economic assessments of other treatment methods for distal radius fractures are largely lacking. No study has investigated cost- effectiveness of distal radius fracture surgery beyond a one-year perspective. The purpose of this study was to assess the cost-effectiveness of VLP versus EF for surgical treatment of patients 50–74 years old with a dorsally displaced distal radius fractures during the first 3 years after distal radius fracture surgery.

## Materials and methods

This study is a cost-utility analysis based on patients included in a previously published randomised controlled trial (RCT) comparing VLP with EF regarding functional outcome [6,9]. Patients eligible were 50–74 years of age with a distal radius fracture of >20 degrees dorsal angulation after a low energetic trauma presenting at a second-level trauma hospital in Stockholm, Sweden, during September 2009 to February 2013. Full inclusion and exclusion criteria are presented in Table 1. 140 patients were randomised through opening of sealed opaque envelopes to EF (Hoffman Compact T2, Stryker, Switzerland) or VLP fixation (2.4 mm Variable Angle LCP Two-Column Volar Distal Radius Plate, Synthes, Switzerland). Data regarding health effect was prospectively collected during the trial period. Cost data was collected retrospectively. The clinical 1-year and 3-year results [6,9] displayed no differences in Patient-Reported Outcome Measures (PROM) after the first 3 months. The analysis was conducted on an intention-to-treat basis. A power calculation was performed for detection of a 10-points difference in the main outcome Disability of the Arm, Shoulder and Hand (DASH) at one year follow-up of the initial RCT. A separate power calculation for the cost-utility outcomes was not conducted.

**Table 1:** Inclusion and exclusion criteria for patients with distal radius fracture for selection to a randomised controlled trial comparing volar locking plate and external fixation.

Inclusion criteria	Exclusion criteria
Patient age (50–74 years for women and 60–74 years for men)	Former disability of either wrist
Injury only after fall from standing height	Other concomitant injuries
Wrist radiography of >20 degree-dorsal angulation and/or >5 mm axial shortening (OTA class 23 A2, A3, C1, C2, C3)	Rheumatoid arthritis or other severe joint disorder
Good knowledge of written and spoken Swedish	Dementia or Pfeiffer score*<5
Fracture diagnosed within 72 hours from injury	Drug or alcohol abuse, or psychiatric disorder
Patient resident within the catchment area of the hospital	Dependency in activities of daily living
	Medical condition contraindicating general anaesthesia

*Adapted from Pfeiffer, E. A short portable mental status questionnaire for the assessment of organic brain deficit in elderly patients. J Am Geriatric Soc. 1975;23:433–441.

### Cost-effectiveness

This cost-utility analysis has been conducted using a health care perspective as well as using a broader perspective including production loss. The time horizons used are one and three years. The primary outcome was the incremental cost-effectiveness ratio (ICER) for VLP compared with EF using a health care perspective including production loss. The ICER was defined as the difference in mean total cost per patient divided by the difference in mean Quality-adjusted-life years (QALY) per patient, expressed as the incremental cost per gained QALY for VLP compared with EF. If the mean difference in QALYs was negative and the mean difference in total cost positive, no ICER was calculated, as VLP then was considered to be dominated by EF. If the mean difference in total cost was negative and the mean difference in QALYs positive, no ICER was calculated, as VLP then was considered to dominate EF. Both costs and QALYs were in line with national guidelines discounted at a discount rate of three percent [10].

### Costs

Total costs per patient were calculated by first identifying and estimating the resource use associated with each surgical method and then valuing each resource using the unit costs presented in Table 2. The direct costs and indirect costs during the first year and up to 3 years for each treatment were summed up.

**Table 2:** Unit costs used in a cost-utility analysis comparing volar locking plate and external fixation in patients with distal radius fractures.

	Unit	Cost (Euro)	Reference
Direct costs	Costs for primary surgery		
	Volar locking plate implant including intraoperative antibiotics, dressings and cast^	441.4	Manufacturers price list
	External fixation implant and dressings^^	122.3	Manufacturers price list
	Operation theatre minute including fixed equipment + overhead costs per minute	2.69	SBU¤
	Operation assistant per minute	0.73	SBU¤
	Surgical nurse per minute	1.08	SBU¤
	Anaesthetic nurse per minute	1.08	SBU¤
	Anaesthesist per minute	2.15	SBU¤
	Orthopaedic surgeon per minute	2.15	SBU¤
	**Costs for reoperations**		
	Carpal ligament release	597.6	*
	Tendon transfer	1049.9	**
	Volar locking plate fixation	1731.4	***
	Volar locking plate extraction/screw extraction	895.4	****
	Soft tissue surgery (fasciotomy, scar release, secondary suture, wound debridement	856.7	*****
	**Costs for hospital care**		
	Emergency visit	375.9	DRG
	Outpatient visit	153.8	DRG
	Day of inpatient care	449.9	DRG
	Occupational therapy, visit	108.4	DRG
	**Costs for X-ray**	51.8	DRG
	**Costs for Drugs**		
	Antibiotics, 1-day use, regular dose	1.53	FASS Drug registry
	Paracetamol, 1-day use, regular dose	0.41	FASS, Drug registry
	Opioids, 1-day use, regular dose	1.91	FASS, Drug registry
	Non-steroid anti-inflammatory drugs, 1-day use, regular dose	0.25	FASS, Drug registry
	Neuroleptics	1.40	FASS, Drug registry
**Indirect costs**	Production loss per day	179.46	SCB

^2.4-mm Variable Angle LCP Two-Column Volar Distal Radius Plate, Synthes, Switzerland.

^^Hoffman Compact T2, Stryker, Switzerland.

¤ **Mellstrand Navarro C, Brolund A, Ekholm C, Heintz E, Hoxha Ekstrom E, Josefsson PO, Leander L, Nordstrom P, Ziden L, Stenstrom K.** Treatment of radius or ulna fractures in the elderly: A systematic review covering effectiveness, safety, economic aspects and current practice. *PLoS One 2019;14–3*:*e0214362*.

*15 min surgical time, 40 min preparation time and 60 min postoperative time in the operation theatre. Operation assistant and surgical nurse attending all time, orthopaedic surgeon attending during surgical time, 10 min before surgery and 10 min after surgery. Dressings.

**45 min surgical time, 40 min preparation time and 60 min postoperative time in the operating theatre. Operation assistant, surgical nurse and anaesthetic nurse attending all time, orthopaedic surgeon attending during surgical time, 10 min before surgery and 10 min after surgery and anaesthesist attending 45 min. Dressings.

***70 min surgical time, 40 min preparation time and 60 min postoperative time in the operating theatre. Operation assistant, surgical nurse and anaesthetic nurse attending all time, orthopaedic surgeon attending during surgical time, 10 min before surgery and 10 min after surgery and anaesthesist attending 45 min. Volar locking plate implant including dressings, cast and one dose of antibiotics. One x-ray.

****25 min surgical time, 40 min preparation time and 60 min postoperative time in the operating theatre. Operation assistant, surgical nurse and anaesthetic nurse attending all time, orthopaedic surgeon attending during surgical time, 10 min before surgery and 10 min after surgery and anaesthesist attending 45 min. Dressings.

*****20 min surgical time, 40 min preparation time and 60 min postoperative time in the operating theatre. Operation assistant, surgical nurse and anaesthetic nurse attending all time, orthopaedic surgeon attending during surgical time, 10 min before surgery and 10 min after surgery and anaesthesist attending 45 min. Dressings.

SBU, the Swedish Agency for Health Technology Assessment and Assessment of Social Services.

DRG, Diagnose-related group financial reimbursement system used at the hospital.

FASS, a compilation from the pharmaceutical industry with information about drugs used in Sweden.

Drug registry, a registry held by the Swedish National Board of Health and Welfare.

### Unit costs

All unit costs are presented in Table 2. Unit costs for operating theatre including staff were derived from a report by the Swedish Agency for Health Technology Assessment and Assessment of Social Services (SBU) [11]. Costs regarding in-and outpatient care including emergency ward visits were collected from the diagnose-related group (DRG [12]) financial reimbursement system used at the hospital. Costs for drug usage were calculated from prices defined in FASS [13] (a compilation from the pharmaceutical industry with information about drugs used in Sweden) for a Defined Daily Dose as defined in the Drug Registry of the Swedish National Board of Health and Welfare [14]. Unit costs for reoperations were calculated based on estimations by the study group regarding surgical time and material usage. All costs above are considered as direct costs. Indirect costs consisted of production loss due to sick leave after the surgery. The unit cost regarding production loss per day was derived from Statistics Sweden [15], using the mean income for adults 20–74 years plus taxes and social services fee. Costs from 2016 were converted to 2018 years prices using a 2% mark-up for every year. All costs are presented in euros converted from Swedish kronor (SEK) with an exchange rate of 0.0978.

### Resource use

All resources needed for each treatment method were identified by the research group. Resource use data for surgical time for the primary surgery was derived from prospectively inserted data in the surgery software system used at the hospital (Orbit [16]). Inpatient and outpatient visits for diagnoses related to the initial injury and any possible related complication (International Classifications of Disease, ICD-10 codes [17] specified in Appendix) were retrieved at an individual level as registry data from the Swedish National Board of Health and Welfare. Drug usage was defined as prescription of antibiotics and analgesics (Anatomical Therapeutic Chemical Classification [18], ATC drug codes specified in Appendix) collected as registry data from the Swedish National Board of Health and Welfare. Data regarding sick leave for diagnoses related to the initial injury and any possible related complication (ICD-10 codes specified in Appendix) were collected as registry data from the Swedish Social Insurance Agency. Any reoperations were detected by search of patient records, and/or registry data retrieval from the Swedish National Board of Health and Welfare regarding surgical procedures related to any possible related complication (NOMESCO classification for surgical procedures codes [19] specified in Appendix). Estimations of resource use were performed by the study group for occupational therapy and x-rays since no complete registry or study protocol source was available. The time frame for all resource use was set to from the date of the injury to the date of the 3-year follow-up.

### Effectiveness

Effectiveness of treatment was estimated using Quality of Life Adjusted Life Years (QALYs) [20,21]. QALYs are a composite measure of survival and Health related Quality of Life, HRQoL. One QALY can be interpreted as the equivalent of one year in full health. The QALYs following each treatment during the study period was calculated on an individual level using the area under the curve (AUC) approach [22]. QALYs for each time interval were calculated by taking the average of the HRQoL at two adjacent time points multiplied with the time in years spent in each time interval. The QALYs gained at 1 year and at 3 years were summarized and an average for each time period was calculated. The HRQoL of the patients was estimated using EuroQol 5 dimensions, EQ-5D-3L [23] and was reported by trial participants at baseline, 2 weeks, 6 weeks, 3 months, 1 year and 3 years postoperatively. EQ-5D-3L is a measure of health status and consists of a questionnaire with five questions and a visual analogue scale (EQ-VAS) [23]. The five questions each represent a dimension of health; mobility, self-care, usual activities, pain/discomfort and anxiety/depression. Each question has three response levels and can be combined into a health profile of five digits [23], which was converted into a health state value using a value set from the United Kingdom (UK) [24].

### Statistical analyses

Data were analysed using SPSS version 26. A complete case analysis was conducted to avoid violating the assumption that data was missing at random, i.e. no imputations were made and only participants with complete data were analysed.

Categorical data was compared with Chi-square test. Normality was tested with Shapiro-Wilks test for all continuous variables. For normally distributed variables independent Student’s t-test was used. Skewed distributed data was compared with Mann-Whitney U-test. Kolmogorov-Smirnov’s test and Kruskal-Wallis´ test were used to confirm statistical significance for non-parametric comparisons. The level of statistical significance was set to p<0.05 in two-sided tests. Linear regression was used to adjust mean differential QALYs at 1 year and 3 years for imbalance between groups in EQ-5D-3L index scores at baseline [25]. Health state values (the EQ-5D-3L index scores in this study) at baseline (before treatment) is often invariably imbalanced between trial arms and it is recommended that the comparisons between treatments are adjusted for this imbalance as it otherwise will contribute to a difference in QALYs that is not an effect of the treatments [25]. Therefore, the difference in mean QALYs between VLP and EF was adjusted for differences in EQ-5D-3L index scores between VLP and EF at baseline (before surgery).

The non-parametric bootstrapping approach with replacement [26] was used to determine the level of sampling uncertainty around the ICER. The bootstrap was performed as a resampling from the original sample to create 1000 random samples. In each bootstrap sample, 58 individuals among the VLP patients and 55 individuals among the EF patients were randomly selected with equal probability and with replacement after each individual selection. To adjust for baseline differences in EQ-5D-3L index scores between the groups [25], we calculated the adjusted differential QALYs (VLP = intervention, EF = control) in each sample.

1000 estimates of incremental costs and effects were generated. The bootstrap is presented in a cost-effectiveness plane [26]. From the bootstrap a cost-effectiveness acceptability curve (CEAC) was derived at 1 year and 3 years, to express the probability that VLP is cost-effective in comparison to EF for a range of thresholds for willingness to pay (WTP) per gained QALY [26].

A threshold of 35000 euros was chosen as maximum WTP per gained QALY, which approximates the 30000 UK pounds sterling used by the National Institute for Health and Clinical Excellence (NICE) in the UK [27].

### Ethics

The conduction of this study was approved by the Regional board for ethical vetting, Stockholm, Sweden, ref nr 2008/1908-31/4, 2009/715-31/2, 2012/2201-32, 2012/1363-32, 2016/2207-32. The collection of data analysed in this trial was recorded at clinicaltrials.gov (NCT 01034943, NCT01035359).

## Results

Of the 140 patients randomised, 6 dropped out before the first year and 16 thereafter, leaving 118 patients for the 3-year follow-up. There were no missing data regarding resource use. Of the 118 patients, five had not filled in all EQ-5D-3L questionnaires and were excluded, leaving 113 patients (VLP n = 58, EF n = 55) for the cost-effectiveness analysis. From the EF group, four patients were converted to VLP intraoperatively and four received a volar plate within the first two weeks after primary surgery, but they were still evaluated within the EF group. Baseline characteristics are presented in Table 3.

**Table 3:** Baseline characteristics of study population in a cost-utility analysis comparing volar locking plate and external fixation in patients with distal radius fractures.

	Volar locking plate (n = 58)	External fixation (n = 55)	P-value
Women (%)	51 (88%)	53 (96%)	0.163*
Age, mean (SD)	63 (6.3)	63 (6.7)	0.460**
Injury to dominant hand	21 (36.2%)	29 (53%)	0.090*
AO-class****			
- A2	3 (5.5%)	3 (5.2%)	0.898***
- A3	19 (34.5%)	15 (25.9%)	
- C1	32 (58.2%)	31 (53.4%)	
- C2	2 (3.6%)	4 (6.9%)	
- C3	2 (3.6%)	2 (3.4%)	

*Chi-square test.

**Student’s t-test.

***Fisher’s exact test.

****Müller ME, Nazarian S, Koch P, The Comprehensive Classification of Fractures of Long Bones, Springer Verlag, Berlin, Heidelberg, 1990.

SD, Standard Deviation.

### Resource utilization and costs

**Fig 1:**
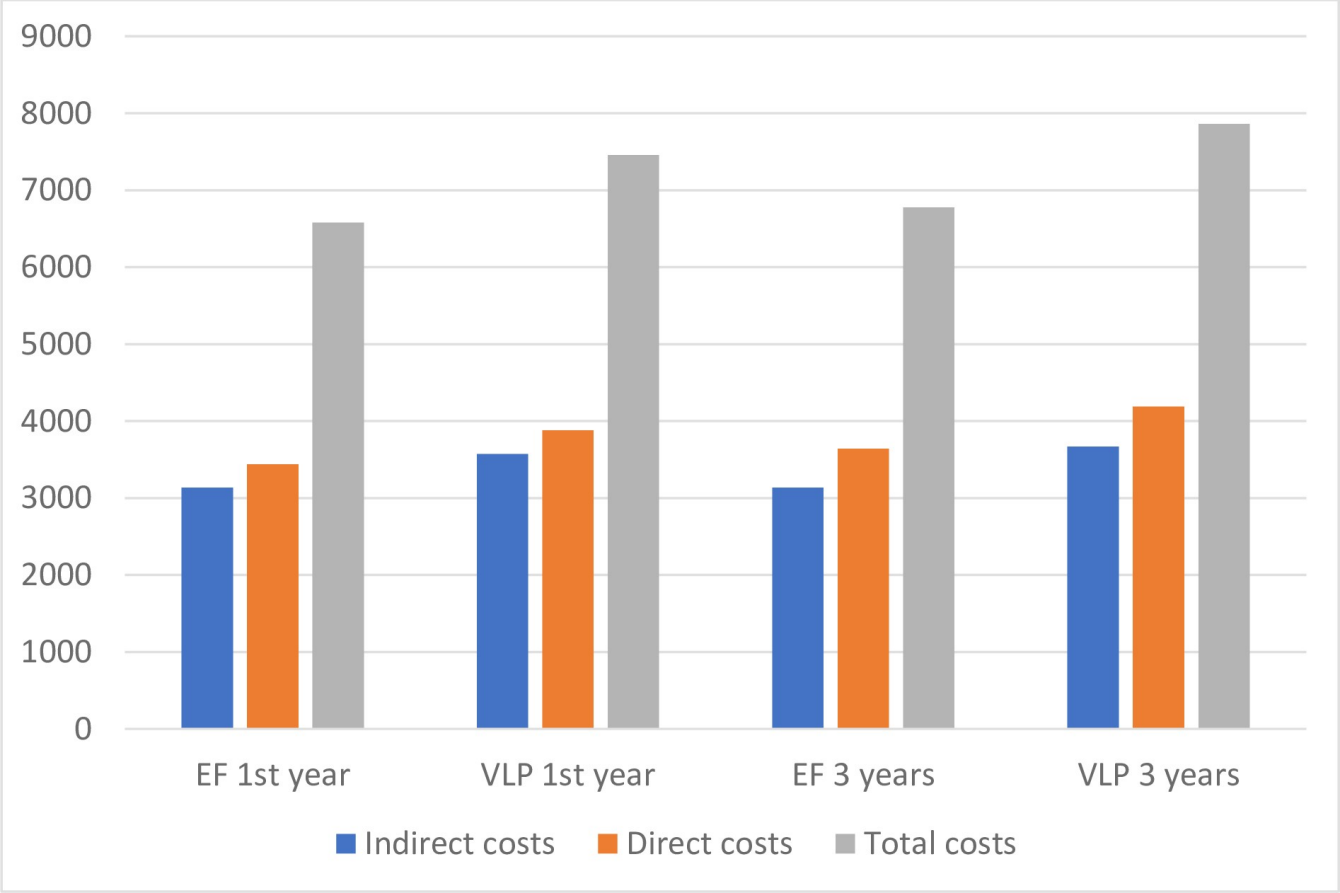
Mean costs for external fixation (EF) patients and volar locking plate (VPL) patients one and three years after distal radius fracture surgery.

Resource utilization is presented in Table 4. All costs are presented in Table 5 and Fig 1. The mean total cost was significantly higher for the VLP group compared with the EF group at 1 year (mean difference; MD: 878 euros, p = 0.006). Mean total cost increased for both groups until the 3-year follow-up, and VLP costs were still significantly higher (MD: 1 082 euros, p = 0.012).

**Table 4:** Resource utilization used in a cost-utility analysis comparing volar locking plate and external fixation in patients with distal radius fractures.

Resource Utilization										
Unit	Volar locking plate (n = 58)				External fixation (n = 55)				Diff (mean)	P-value
	Mean (SD)	Min, Max	Median	Missing (%)	Mean (SD)	Min, Max	Median	Missing (%)		
**Primary surgery**										
Time in operating theatre (min)	70 (18)	36–113	68	0	43 (24)	19–135	35	0	27	<0.001
Preparing time + postoperative time in operating theatre (min)	40 + 60				40 + 60					
**Reoperations**										
Carpal ligament release,										
- 1st year	0.02 (0.13)	0–1	0.0	0	0.02 (0.14)	0–1	0.0	0	-0.001	0.970
- 3 years	0.02 (0.13)	0–1	0.0	0	0.02 (0.14)	0–1	0.0	0	-0.001	0.970
Tendon transfer										
- 1st year	0.02 (0.13)	0–1	0.0	0	0.0		0.0	0	0.017	0.330
- 3 years	0.02 (0.13)	0–1	0.0	0	0.0		0.0	0	0.017	0.330
Volar locking plate fixation										
- 1st year	0.02 (0.13)	0–1	0.0	0	0.05 (0.23)	0–1	0.0	0	-0.037	0.286
- 3 years	0.02 (0.13)	0–1	0.0	0	0.05 (0.23)	0–1	0.0	0	-0.037	0.286
Plate extraction										
- 1st year	0.09 (0.28)	0–1	0.0	0	0.04 (0.19)	0–1	0.0	0	0.05	0.274
- 3 years	0.17 (0.38)	0–1	0.0	0	0.04 (0.19)	0–1	0.0	0	0.13	0.019
Soft tissue surgery (fasciotomy, scar release, secondary suture, wound debridement										
- 1st year	0.07 (0.53)	0–4	0.0	0	0.05 (0.05)	0–1	0.0	0	0.014	0.298
- 3 years	0.09 (0.54)	0–4	0.0	0	0.05 (0.05)	0–1	0.0	0	0.032	0.623
**Hospital care**										
Emergency visits	1				1					
Outpatient visits										
- 1st year	5.1	4–9	5	0	5.7 (1.5)	2–11	5	0	-0.6	0.005
- 3 years	6.4	4–14	6	0	6.8 (6.0)	3–13	6	0	-0.4	0.165
Inpatient care days										
- 1st year	0.5 (1.9)	0–10	0	0	0.2 (0.7)	0–4	0	0	0.3	0.974
- 3 years	0.5 (1.9)	0–10	0	0	0.2 (0.7)	0–4	0	0	0.3	0.974
Occupational therapy visits										
- 1st year	4	4	4	0	5	5	5	0	-1	Not
- 3 years	4	4	4	0	5	5	5	0	-1	relevant**
**X-ray**	2	2	2	0	2	2	2	0	0	Not relevant**
**Drugs, daily doses**										
Antibiotics										
- 1st year	1.0 (4.3)	0–25.0	0.0	0	6.4 (20.0)	0–113.0	0.0	0	-5.4	0.099
- 3 years	3.4 (8.4)	0–37.5	0.0	0	8.3 (23.5)	0–123.8	0.0	0	-4.9	0.746
Paracetamol										
- 1st year	21.4 (55.9)	0–291.7	0.0	0	16.1 (35.9)	0–200.0	0.0	0	5.3	0.952
- 3 years	44.0 (110.0)	0–641.8	0.0		61.5 (143.8)	0–678.1	0.0	0	-17.5	0.789
Opioids										
- 1st year	32.0 (32.9)	0–206.0	25.7	0	27.9 (25.5)	0–117.0	23.3	0	4.2	0.539
- 3 years	39.4 (49.1)	0–299.1	32.7	0	39.6 (78.5)	0–575.2	23.3	0	-0.2	0.495
Non-steroid anti-inflammatory drugs										
- 1st year	16.8 (67.8)	0–423.3	0.0	0	8.0 (26.7)	0–160.0	0.0	0	8.8	0.843
- 3 years	49.3 (173.1)	0–1212.5	0.0	0	29.5 (65.7)	0–320.0	0.0	0	19.8	0.669
Neuroleptic drugs										
- 1st year	1.3 (7.3)	0–50.0	0	0	0	0	0	0	1.3	0.167
- 3 years	1.6 (8.3)	0–58.3	0	0	0	0	0	0	1.6	0.089
**Sick leave (days**)										
- 1st year	19.9 (46)	0–259	0	0	17.5 (32)	0–114	0	0	2.4	0.650
- 3 years	20.5 (49	0–291	0	0	17.5 (32)	0–114	0	0	3.0	0.650

*Student’s t-test.

** Estimations of resource use were performed by the study group since no complete registry or study protocol source was available.

**Table 5:** Costs in euros used in a cost-utility analysis comparing volar locking plate and external fixation in patients with distal radius fractures.

	Volar locking plate Mean (SD)	External fixation Mean (SD)	Diff (mean)	p-value*
Implant	441.4 (0)	145.4 (86.6)	295.9	<0.001
Operation theatre	456.8 (47.5)	385.4 (65.3)	71.4	<0.001
Operation staff	780.2 (89.0)	646.5 (604.7)	133.7	<0.001
**Total cost for primary surgery**	1678.3 (136.5)	1177.4 (260.8)	500.9	<0.001
**Reoperations**				
Carpal ligament release				
- 1st year	10.3 (78.5)	10.9 (80.6)	-0.6	0.970
- 3 years	10.3 (78.4)	10.9 (80.6)	-0.6	0.970
Tendon transfer				
- 1st year	18.1 (137.9)	0	18.1	0.330
- 3 years	18.1 (137.9)	0	18.1	0.330
Volar locking plate fixation				
- 1st year	29.9 (227.4)	94.4 (396.8)	-64.6	0.286
- 3 years	29.9 (227.4)	94.4 (396.8)	-64.6	0.286
Volar locking plate extraction				
- 1st year	77.2 (253.5)	32.6 (169.1)	44.6	0.274
- 3 years	152.1 (336.2)	32.6 (169.1)	119.6	0.023
Soft tissue surgery				
- 1st year	59.1 (450.0)	46.7 (196.3)	12.4	0.298
- 3 years	73.4 (461.2)	46.7 (196.3)	26.7	0.606
All reoperations				
- 1st year	194.5 (608.6)	184.6 (516.1)	9.9	0.962
- 3 years	283.8 (726.7)	184.6 (516.1)	99.2	0.358
**Hospital care**				
Outpatient care including primary emergency visit				
- 1st year	1161.1 (140.3)	1254.2 (228.7)	-93.2	0.005
- 3 years	1347.4 (255.2)	1408.3 (284.7)	-60.8	0.101
Inpatient care				
1st year	232.7 (831.0)	106.3 (311.7)	126.4	0.974
3 years	232.7 (831.0)	106.3 (311.7)	126.4	0.974
Occupational therapy				Not relevant**
- 1st year (4432/5540)	433.4	541.8	-108.4	
- 3 years (4432/5540)	433.4	541.8	-108.4	
**X-ray**				Not relevant**
- 1st year	103.7	103.7	0	
- 3 years	103.7	103.7	0	
**Drugs**				
Antibiotics				
- 1st year	1.55 (6.58)	9.79 (30.51)	-8.2	0.099
- 3 years	5.06 (12.38)	12.56 (35.58)	-7.5	0.743
Paracetamol				
- 1st year	8.79 (22.96)	6.61 (14.75)	2.2	0.952
- 3 years	17.63 (44.14)	24.43 (56.91)	-6.8	0.751
Opioids				
- 1st year	61.12 (62.81)	53.20 (48.58)	7.9	0.539
- 3 years	74.45 (91.95)	74.46 (143.58)	-0.01	0.482
Non-steroid anti-inflammatory drugs				
- 1st year	4.11 (16.58)	1.96 (6.53)	2.1	0.843
- 3 years	11.72 (41.22)	7.02 (15.65)	4.7	0.677
Neuroleptics				
- 1st year	1.81 (10.19)	0	1.8	0.167
- 3 years	2.19 (11.60)	0	2.2	0.089
All drugs				
- 1st year	77.38 (94.28)	71.56 (62.58)	5.8	0.968
- 3 years	111.05 (161.66)	118.47 (193.55)	-7.4	0.859
**Total direct costs**				
- 1st year	3881 (1439)	3440 (897)	442	**<0.001**
- 3 years	4190 (1640)	3641 (921)	550	**<0.001**
**Indirect costs**				
Sick leave				
- 1st year	3575 (8261)	3138 (5677)	436	0.650
- 3 years	3671 (8785)	3138 (5677)	533	0.650
**Total cost (direct and indirect costs)**				
- 1st year	7456 (8329)	6578 (5745)	878	**0.006**
- 3 years	7861 (9011)	6778 (5733)	1082	**0.012**

*Mann-Whitney U-test.

**Estimations of resource use were performed by the study group since no complete registry or study protocol source was available.

### Health-related quality of life

**Fig 2:**
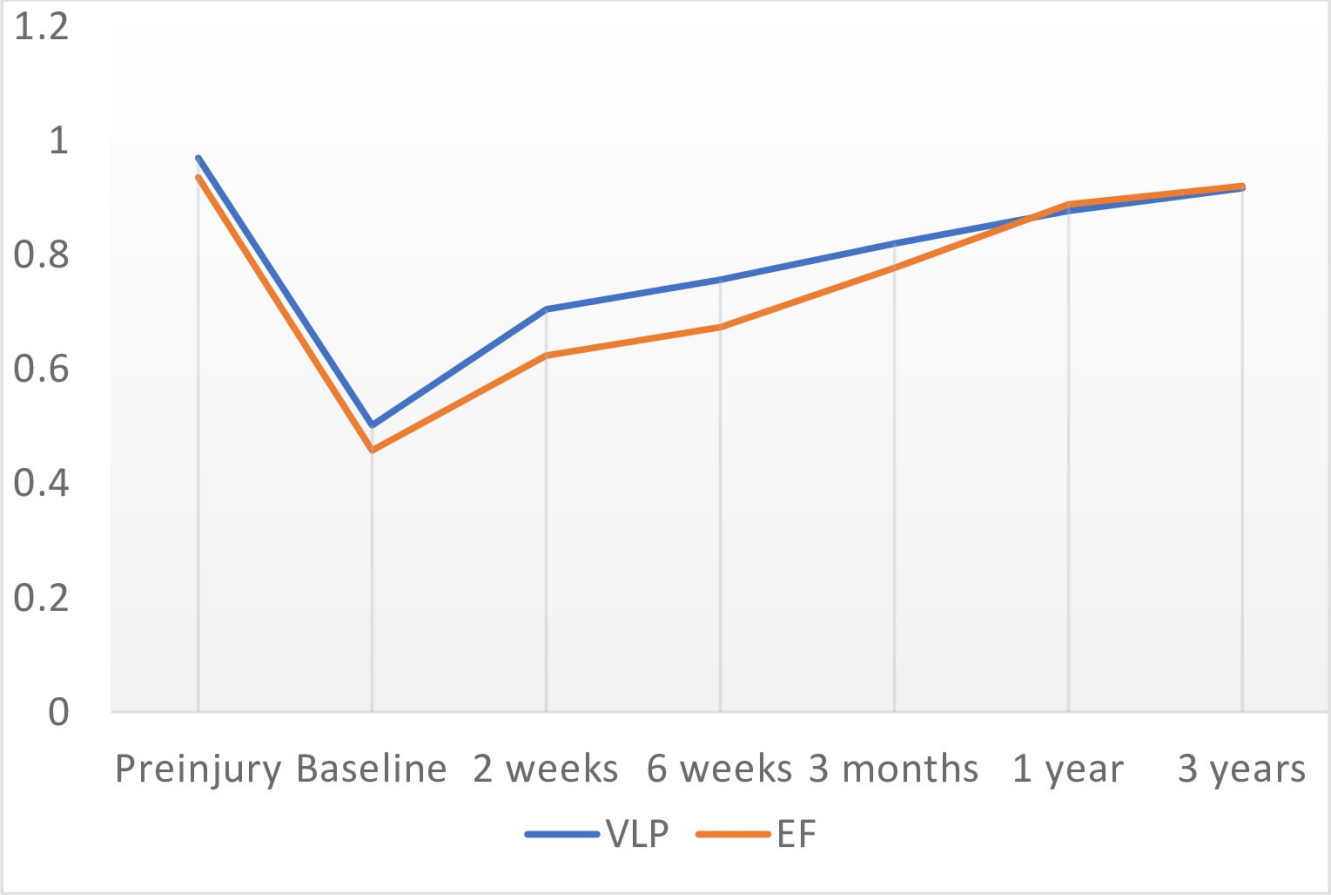
Mean EQ-5D-3L index scores at preinjury, baseline and follow-up points after surgery with volar locking plate (VLP) and external fixation (EF).

EQ-5D-3L index scores are presented in Table 6 and Fig 2. At 2 and 6 weeks, the VLP group had statistically significant better EQ-5D-3L index scores than the EF group, but differences did not remain at later follow-up time points. Mean EQ-5D-3L index scores improved continuously between all timepoints but was still lower than pre-injury levels at 3 years. Mean total QALYs during the first year was 0.814 in the VLP group and 0.787 in the EF group (p = 0.236) (Table 7). After adjustments for baseline differences in EQ-5D-3L index scores between the groups, the difference in mean total QALYs was 0.020 (p = 0.344) in favor of the VLP group. At 3 years, mean total QALYs was 2.530 in the VLP group and 2.518 in the EF group (p = 0.852). The adjusted mean difference was 0.005 (p = 0.932) in favor of the EF group (Table 7).

**Table 6:** Mean EQ-5D-3L index scores at pre-injury, baseline and follow-up points after distal radius fracture surgery with volar locking plate and external fixation.

EQ-5D-3L index score	Volar locking plate Mean (SD)	External fixation Mean (SD)	p-value*
Pre-injury	0.970 (0.076)	0.936 (0.129)	0.104
Baseline	0.502 (0.278)	0.458 (0.317)	0.652
2-week follow-up	0.705 (0.197)	0.624 (0.217)	**0.018**
6-week follow-up	0.757 (0.189)	0.674 (0.208)	**0.009**
3-month follow-up	0.820 (0.112)	0.777 (0.176)	0.158
1-year follow-up	0.877 (0.189)	0.889 (0.132)	0.766
3-year follow-up	0.917 (0.132)	0.921 (0.131)	0.852

*Mann-Whitney U-test.

**Table 7:** Cost-utility analysis for volar locking plate fixation (VLP) compared to external fixation (EF) after distal radius fracture surgery.

	Costs (Euro) 1^st^ year	QALYs 1^st^ year	Cost per QALY gained 1^st^ year	Costs (Euro) 3 years	QALYs at 3 years	Cost per QALY gained at 3 years
**Health care perspective**						
VLP	3881	0.814	**22 100**	4190	2.5302	**Dominated**
EF	3440	0.787		3641	2.5181	
Difference	**442**	0.020*		**550**	-0.005*	
Health care perspective plus production loss						
VLP	7456	0.814	**43 900**	7861	2.5302	**Dominated**
EF	6578	0.787		6778	2.5181	
Difference	878	0.020*		1082	-0.005*	

*Adjusted for baseline differences.

### Cost-utility analysis

**Fig 3:**
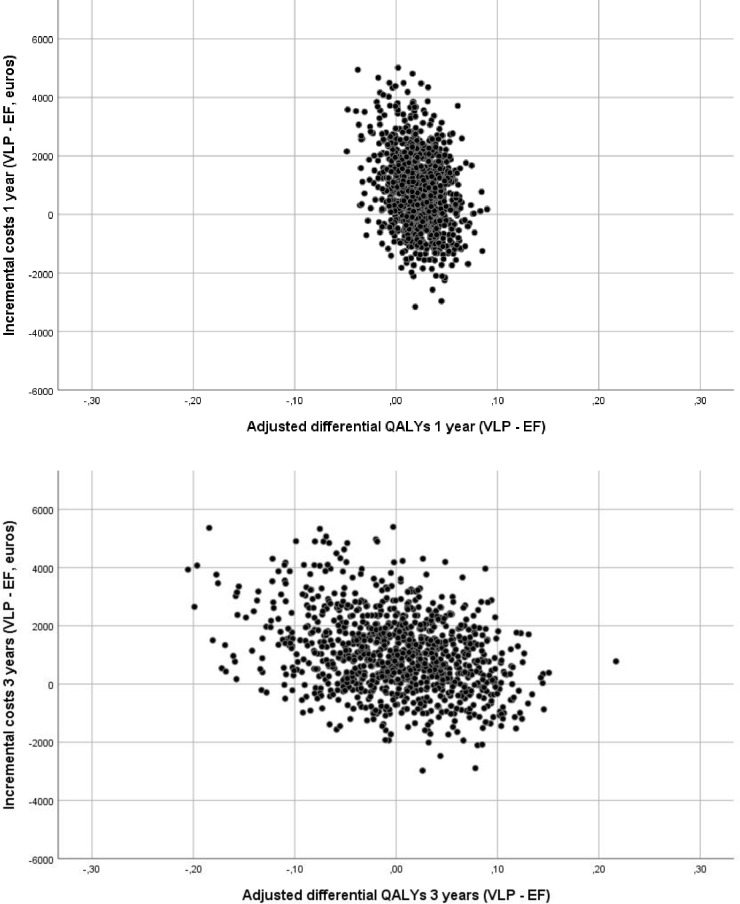
Scatterplots of 1 000 samples of bootstrapped differences in mean costs and quality adjusted life years (QALYs) (adjusted for baseline difference in EQ-5D-3L index scores) over one year and three years after volar locking plate (VLP) compared to external fixation (EF), in cost-effectiveness planes.

**Fig 4:**
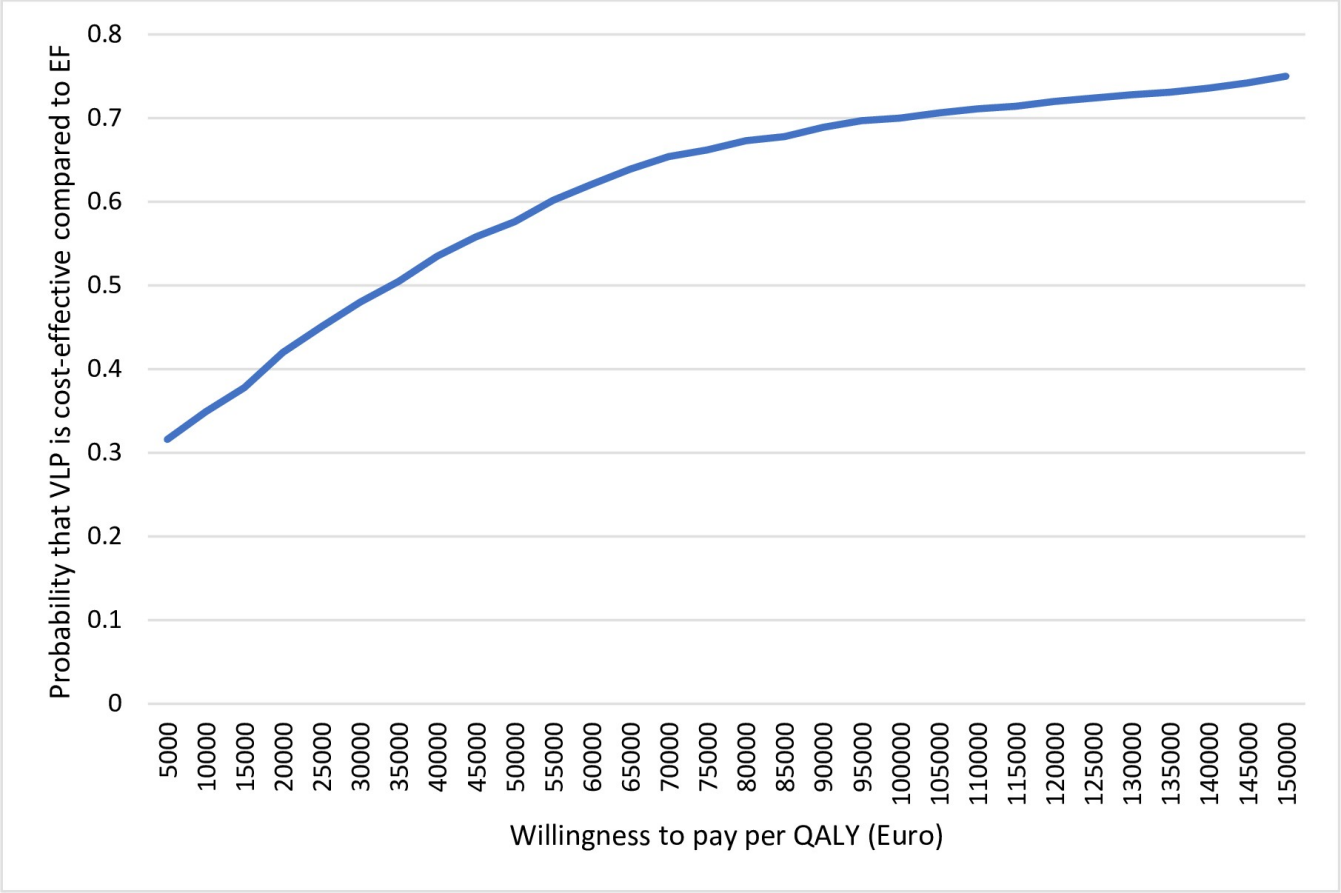
Cost-effectiveness acceptability cure (CEAC) representing the probability of the cost-effectiveness of treatment using a volar locking plate (VLP) compared with external fixation (EF) at different willingness to pay (WTP) thresholds at one year after distal radius fracture surgery.

**Fig 5:**
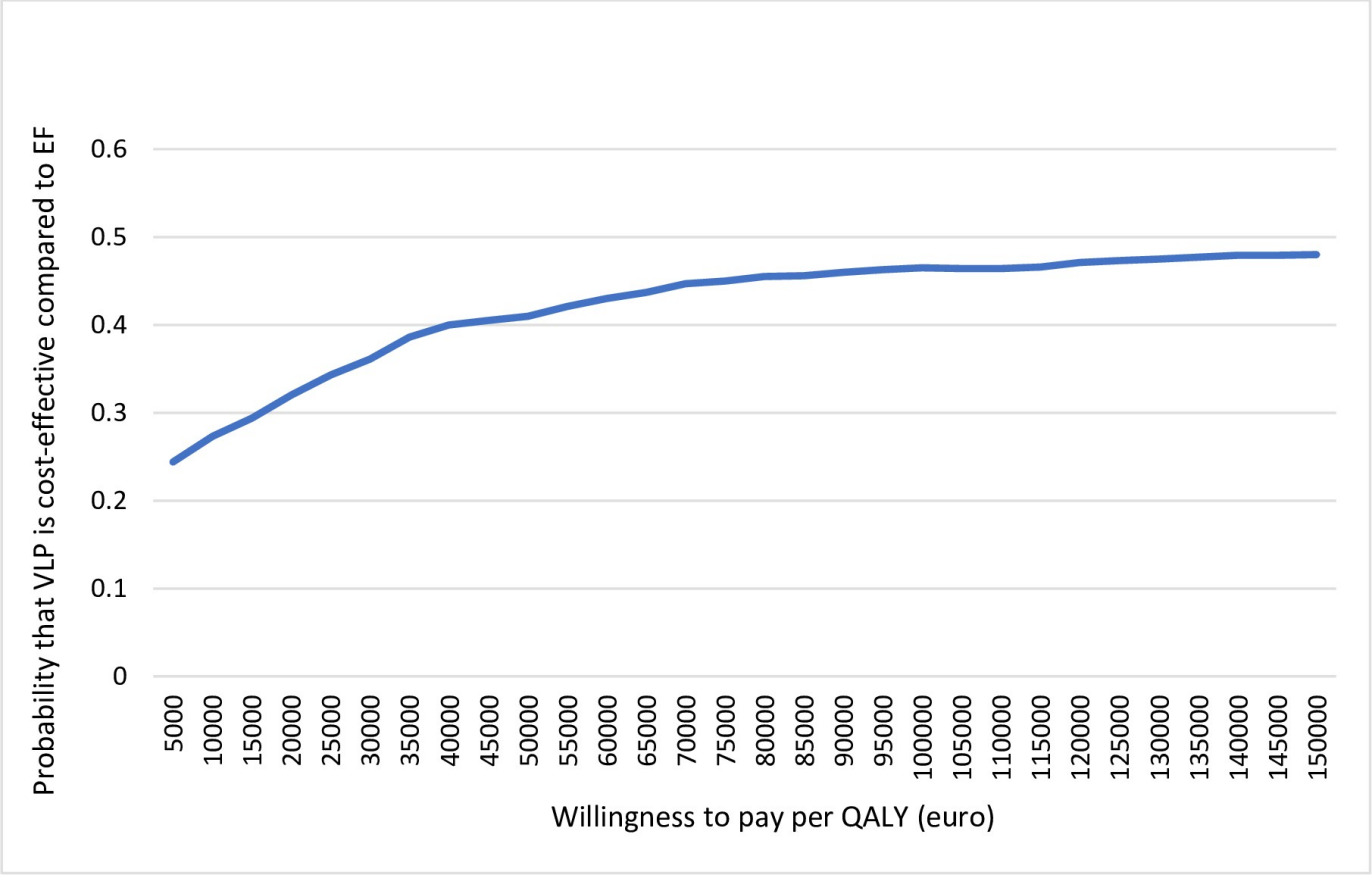
Cost-effectiveness acceptability cure representing the probability of the cost-effectiveness of treatment using a volar locking plate (VLP) compared with external fixation (EF) at different willingness-to-pay thresholds at three years after distal radius fracture surgery.

From a health care perspective, the ICER at 1 year was 22 100 euros per QALY for VLP fixation compared to EF (Table 7). When including production loss, the ICER increased to 43 900 euros per QALY. At 3 years, VLP resulted in higher costs and a smaller effect in QALYs than EF, independent of whether production loss was included or not. This means that VLP was dominated by EF in the longer time horizon. The bootstrap analyses of the estimates including production loss are presented in cost-effectiveness planes (Fig 3). The scatterplot covers all four quadrants indicating uncertainty about whether or not VLP is cost-effective and at what value it is cost-effective compared to EF. The Cost Effectiveness Acceptability curves (CEAC) in Figs 4 and 5 summarize the probability of VLP being cost-effective compared to EF at one and three years respectively. At a willingness to pay threshold of 35 000 euros per QALY, the probability that VLP is cost-effective compared to EF is around 50% at 1 year and 40% at 3 years. At 3 years, the probability that VLP is cost-effective does not exceed 50% independent of the willingness to pay per QALY.

## Discussion

The purpose of this study was to assess the cost-effectiveness of VLP compared to EF. The study shows that at 3 years, VLP patients had higher costs and a smaller effect (although not statistically significant) in QALYs compared to EF patients independent of the perspective used, indicating that VLP is not cost-effective compared to EF. At 1 year, the VLP group had gained more QALYs than the EF group, and the incremental total cost per QALY gained for the health care perspective (excluding production loss) was below the threshold recommended by NICE. However, when including production loss, the threshold was exceeded. Between 1 year and 3 years, VLP patients increased their costs more than EF patients and EF patients increased their EQ-5D-3L index scores more than VLP patients. The statistical analyses displayed a high level of uncertainty surrounding the ICER, which implies that further studies are needed to support our findings.

There are no previous cost-utility studies comparing VLP with EF, but there are some studies comparing VLP with percutaneous pinning. Tubeuf et al [8] found a statistically significant incremental cost of 815 euros (converted from UK pounds sterling) after one year for VLP compared with percutaneous pinning. As VLP patients had a smaller gain in QALYs (0.008) than in our study, the resulting ICER was higher (100 295 euros per QALY). However, they did not investigate patients beyond the first year. Karantana et al [7] presented a study comparing VLP with percutaneous pinning and optional EF (11/64 patients) and showed a statistically significant incremental cost of 801 euros (converted from UK pounds sterling) after 1 year. They also presented a smaller gain in QALYs (0.0178) for VLP patients than our study, resulting in an ICER of 44 990 euros per QALY for the VLP group in comparison with the percutaneous pinning group.

Differences in EQ-5D-3L index scores and resulting QALYs were very small in the studies of Tubeuf [8] and Karantana [7], which is in accordance with the findings in our study. Even small differences in total costs render large differences in ICER due to small differences in QALYs. As VLP was associated with higher costs, VLP would still not be considered cost-effective even if there were no differences in QALYs.

The major strength of the present study is the relatively long follow-up period as treatment-related costs still occur after the first year, and HRQoL continues to improve. Another strength is that the study is conducted within the scope of a randomised trial, thus decreasing the risk of an impact on the results of potential biases. One strength is also that we have used registry data from the Swedish National Board of Health and Welfare, thereby capturing any resource use occurring at other hospitals or care providers. The use of registry data is also a limitation as we searched for ICD-10 codes and drug prescriptions that we assumed could be associated with the distal radius fracture, possibly rendering an overestimation of outpatient visits and drug usage. Another limitation is that we could not, in the retrospective perspective, evaluate the resource use of occupational therapy and x-rays and therefore had to make an estimation. Moreover, there was no data on primary care or nurse visits. Lastly, the study population is relatively small, thus limiting the power of detecting small differences between groups.

In conclusion, VLP fixation was associated with higher costs and resulted in fewer QALYs gained compared to EF at 3 years after distal radius fracture surgery., At this time horizon, the probability of VLP being cost-effective as compared to EF did not exceed 50% when including production loss, independent of the willingness to pay per QALY when adaption a perspective including production loss. Thus, our results indicate that it is uncertain if VLP is a cost-effective treatment of unstable distal radius fractures compared to EF.

## Supporting information

S1 File A CHEERS checklist. (PDF)
